# Formative research to design a culturally-appropriate cancer clinical trial education program to increase participation of African American and Latino communities

**DOI:** 10.1186/s12889-020-08939-4

**Published:** 2020-06-03

**Authors:** Jennifer Cunningham-Erves, Claudia Barajas, Tilicia L. Mayo-Gamble, Caree R. McAfee, Pamela C. Hull, Maureen Sanderson, Juan Canedo, Katina Beard, Consuelo H. Wilkins

**Affiliations:** 1 Department of Internal Medicine, Meharry Medical College, 1005 Dr. D. B. Todd Jr. Blvd, Nashville, TN 37208 USA; 2 Vanderbilt Ingram Cancer Center, Vanderbilt University Medical Center, Nashville, TN USA; 3 Department of Health Policy and Community Health, Georgia Southern University, Statesboro, GA USA; 4 Division of Epidemiology, Department of Medicine, Vanderbilt University Medical Center, Nashville, TN USA; 5 Department of Family and Community Medicine, Meharry Medical College, Nashville, TN USA; 6 Progreso Community Center, Nashville, TN USA; 7 School of Graduate Research Studies, Meharry Medical College, Nashville, TN USA; 8 Matthew Walker Community Health Center, Nashville, TN USA; 9 Meharry Vanderbilt Alliance, Nashville, TN USA; 10 VUMC Office of Health Equity, Vanderbilt University Medical Center, Nashville, TN USA

**Keywords:** Cancer, Disparities, Clinical trials, African Americans, Latinos, Education, Recruitment, Community health educators (CHEs)

## Abstract

**Background:**

Addressing knowledge deficiencies about cancer clinical trials and biospecimen donation can potentially improve participation among racial and ethnic minorities. This paper describes the formative research process used to design a culturally-appropriate cancer clinical trials education program for African American and Latino communities. We characterized community member feedback and its integration into the program.

**Methods:**

We incorporated three engagement approaches into the formative research process to iteratively develop the program: including community-based organization (CBO) leaders as research team members, conducting focus groups and cognitive interviews with community members as reviewers/consultants, and interacting with two community advisory groups. An iterative-deductive approach was used to analyze focus group data. Qualitative data from advisory groups and community members were compiled and used to finalize the program.

**Results:**

Focus group themes were: 1) Community Perspectives on Overall Presentation; 2) Community Opinions and Questions on the Content of the Presentation; 3) Culturally Specific Issues to Participation in Cancer Clinical Trials; 4) Barriers to Clinical Trial Participation; and 5) Perspectives of Community Health Educators. Feedback was documented during reviews by scientific experts and community members with suggestions to ensure cultural appropriateness using peripheral, evidential, linguistic, sociocultural strategies, and constituent-involving. The final program consisted of two versions (English and Spanish) of a culturally-appropriate slide presentation with speaker notes and videos representing community member and researcher testimonials.

**Conclusions:**

Incorporating multiple community engagement approaches into formative research processes can facilitate the inclusion of multiple community perspectives and enhance the cultural-appropriateness of the programs designed to promote cancer clinical trial participation among African Americans and Latinos.

## Background

Cancer remains a public health threat as the second leading cause of death nationally and worldwide [1]. Minorities continue to disproportionately share the burden [2]. African Americans have the highest cancer death rates and shortest survival rates for most cancers. While cancer death rates are lower among Latinos compared to most racial/ethnic groups, cancer is the leading cause of death for this group [3]. Participation of racial/ethnic minorities in cancer clinical trials and biospecimen-based research is necessary to be able to identify and test prevention, detection, and treatment methods that are effective for these groups. Yet, research participation of racial/ethnic minorities is alarmingly low. For example, about 3 % of all adult cancer patients enroll in clinical trials [4], and participation is lower among minorities compared to Whites, with minorities representing only 2 % of cancer clinical trial participants but 40% of the U.S. population [4, 5]. Consequently, 20% of studies fail to be completed due to insufficient enrollment [6, 7], impeding advancement of prevention and treatment methods [8]. For studies that do succeed, yet have low minority participation, there is uncertainty of whether the medical interventions can be generalized to different racial/ethnic groups to know whether they will work equally well in these groups [9, 10]. Improving clinical trial participation and biospecimen donation in racial/ethnic minority groups would increase diversity in participation and enable the development of medical interventions that are effective across racial/ethnic groups or need to be modified for specific groups [10].

Barriers to clinical trial participation for African Americans and Latinos are numerous, complex, and some even insurmountable [8, 11]. Examples include low patient trust in physicians and the research process, fear, and systemic social inequalities [12–15]. Lack of awareness and knowledge about research and opportunities for participation are among the strongest barriers [14, 16–19]. Clinical trial education for patients and their families, clinic staff, communities, and institutions have shown promise to help identify, accrue, and retain all trial participants [16, 20–27]. However, these interventions yield inconsistent results. For example, a biospecimen education intervention using a community based participatory research approach demonstrated fewer negative associations with biospecimen participation; however, participation remained unchanged [26]. More research is needed to identify the various factors contributing to patient and community knowledge deficiencies on clinical trials in order to develop targeted educational programs that can potentially improve participation outcomes.

Targeting health communication maximizes the “fit” of information by customizing an educational program to a subgroup of people with unique characteristics [28]. Application of targeting in the development of educational programs has improved many preventive health behaviors (e.g., cancer screening) [29]. Targeting is described as “a single program for a defined population subgroup that takes into account characteristics shared by the subgroups‘ members and increases cultural sensitivity of health programs” [30]. Kreuter et al. [31] recommend using peripheral, evidential, linguistic, sociocultural, and constituent-involving strategies to develop targeted programs to achieve cultural appropriateness [31]. Peripheral strategies increase the appeal of communication through the title, fonts, colors, and/or images. Evidential strategies provide evidence or data on the impact of a health issue on a certain group. Linguistic strategies fit the program to the native language of a certain group, which could involve translations of content. Sociocultural strategies address health issues from the social and cultural values of a certain group. Last, constituent-involving strategies ensure community members inclusion in program planning, decision-making, and/or staff [31].

Formative research combines observational data collection and analysis with program development in an iterative process, such that the data analysis guides the program development rather than answering specific a priori research questions [32, 33]. Formative research is commonly used to develop intervention programs, and it can particularly be used to develop targeted programs to ensure cultural sensitivity [33–35]. It involves the use of quantitative and/or qualitative methods to understand knowledge gaps and social norms of a cultural group, identify behaviors to be addressed, and inform strategies, messages, and channels of communication [33, 36]. The goal of formative research is to learn and incorporate the culture of the target community, build trust, encourage academic-community partnerships, and promote program acceptance [33].

The application of community-engaged research (CEnR) in the formative research process offers the potential to optimize the likelihood that the intervention will have its intended effect [26, 27, 37]. Community engagement is defined as the collaboration of groups to identify and address issues affecting their well-being [38]. The level of community involvement can range along a continuum, ranging from outreach to shared leadership [38], with the goals of the engagement effort aligning with those of formative research. Little guidance is currently available in the scientific literature on how to apply CEnR approaches in a formative research process to improve incorporation of cultural information into an educational program, in particular for educational programs aimed to increase participation of African Americans and Latinos in cancer clinical trials and biospecimen donation for research.

### Preliminary work

The Meharry-Vanderbilt-TSU Cancer Partnership (MVTCP) was formed in 1999 between Meharry Medical College (MMC) and Vanderbilt-Ingram Cancer Center (VICC) and added Tennessee State University (TSU) to the partnership in 2006. The MVTCP Cancer Outreach Core fosters community-academic partnerships, conducts outreach activities, and facilitates CEnR related to cancer disparities. In 2009, the core and six community partners serving local African American, Latino, and rural communities conducted six town halls (*N* = 96). A town hall is a grop session that is conversational in nature that gains the community’s input on health research priorities [39]. The community members discussed cancer clinical trial participation and strategies to overcome barriers to participation among racial/ethnic minorities and medically underserved communities. Those engaged were African American (75%), Latino (14%), rural White (6%), and other/not reported (5%). Most (65%) had not participated in biomedical research. Two emerging themes were distrust and uncertainty in medical research. Suggestions to reduce distrust were to improve communication between research institutions and the community, conduct research on health issues important to the community, and provide clinical trial education programs in the community.

Using this feedback, the partners sought to increase community awareness and local support for cancer clinical trials. VICC then collaborated with the Education Network to Advance Cancer Clinical Trials (ENACCT), a national organization, to use one of its educational programs- entitled, “What Are Cancer Clinical Trials and Why Are They Important for My Community?” [22, 27] Cancer clinical trial topics included types, phases, risks and benefits, and protections. The partners made minor modifications to the program to adapt it for the local communities (i.e., different background and color scheme, inclusion of local statistics on cancer, added logos for partners and contact information for local resources, changed some images and wording, and changed some content for cultural appropriateness). ENACCT provided a 1.5-day, train-the-trainer program to community health educators (CHEs) (4 African American, 2 Latino, 1 White) volunteering from the partner organizations. From 2010 to 2011, the CHEs implemented 17 60-min, educational sessions with 245 participants (130 African American, 46 Latino, and 69 White) (unpublished observations). While the idea for this program initiated from the community in the town halls, a major limitation was the lack of African American and Latino community members’ involvement in the development of the original ENACCT program. The partners concluded that further research was needed to ensure the program: 1) was culturally-appropriate, and 2) addressed common audience questions and concerns.

### Purpose of formative research

This paper describes the formative research process used to design a single-session, in-person educational program aimed to increase participation in cancer clinical trials and biospecimen donation. Our primary goal was to collect data that could be used to develop an effective, culturally-appropriate program for African American and Latino communities. To ensure the program was culturally-appropriate, we used peripheral, evidential, linguistic, and sociocultural strategies to design the program using the feedback. We also used a constituent-involving strategy (i.e., community engagement) to: (1) engage community members in all research phases of program development; and (2) incorporate community feedback into the program.

## Methods

**Fig. 1:**
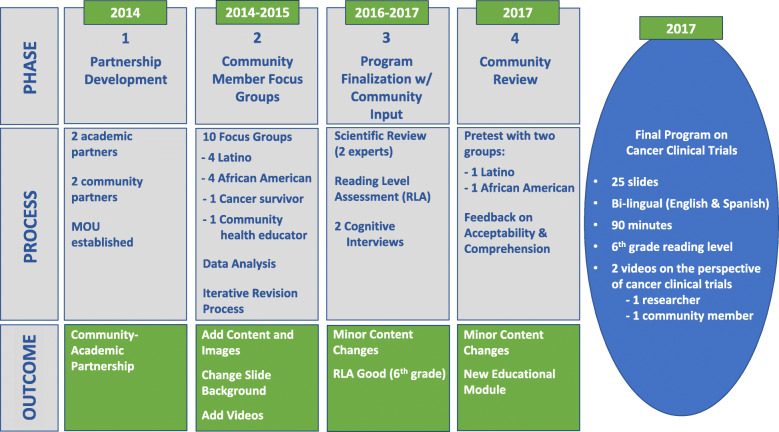
Community-engaged cancer clinical trial program adaptation process

We used the modified ENACCT module described under Preliminary Work as a starting point to gather community input. The initial plan was to adapt this existing program; however, the formative research process and community engagement led to the generation of nearly all new content, which resulted in a new educational program. Our team iteratively designed the new program through a community-engaged, formative research process over 3 years. The formative research process consisted of four phases of data collection and program development while gaining feedback through community participation at each phase. Figure 1 depicts the timeline of the phases to develop the final, new program. This research was approved by Vanderbilt University Institutional Review Board.

### Phase 1: partnership development, 2014

Two community organizations, Matthew Walker Comprehensive Health Center (MWCHC, lead organization of the Nashville Health Disparities Coalition, primarily serving African Americans) and Progreso Community Center (PCC, lead organization of the Nashville Latino Health Coalition, primarily serving Latinos) entered a partnership with the Meharry-Vanderbilt Alliance (MVA) and the MVTCP Cancer Outreach Core to develop the cancer clinical trial educational module based on direct input from African American and Latino communities. We continued use of a CEnR approach throughout this research process to: 1) promote a successful, collaborative process; and 2) ensure the formative research goal was met. Partners met during and throughout each phase (i.e., monthly on average) to make changes to the program iteratively and collectively using community feedback.

### Phase 2: community-member focus groups, 2014–2015

#### Study design and setting

The focus group phase of the formative research process used an observational qualitative study design. We conducted qualitative focus groups to inform new content for the educational module, specifically to gather and analyze data regarding the perception, acceptability, and concerns with content. As a part of the community-engaged process, we started with the modified ENACCT clinical trial education module that the team used previously and iteratively adapted the content into a new module based on the focus group data from African American and Latino community members in Nashville, Tennessee.

#### Sampling and recruitment

A purposive sampling method was used to select participants. Partnership representatives identified community members or other community-based organizations to recruit participants based on the eligibility criteria below. Ten focus groups represented African Americans (*n* = 4), Latinos (n = 4), cancer survivors (*n* = 1), and the CHEs who were previously trained and delivered the modified ENACCT module (n = 1), as described under Preliminary Work. The CHEs were included so they could describe their experience in teaching the material, the challenges they encountered, and the feedback they received from community members to improve content and program delivery. Recruitment methods were flyers, word-of-mouth, and community-based organizations (CBOs).

#### Eligibility criteria

Inclusion criteria were: 1) female or male; 2) English-speaking and Spanish/English speaking if Latino; 3) African American or Latino; and 4) age 18 and older. The cancer survivor group had additional inclusion criteria of being a cancer survivor. The CHE group had additional inclusion criteria of having conducted at least one educational session in the previous phase of the project.

#### Procedures

First, a research team member who self-identified as African American or Latino (congruent to the race/ethnicity of focus group members) presented the modified ENACCT educational module that was used in the previous phase of the project (see Preliminary Work). Next, a focus group discussion was moderated by one of the research team members. Using the literature and our past research, the moderator’s guide queried barriers to research and program cultural appropriateness (e.g., comprehension, delivery effectiveness, relevance) (See Supplementary Material for Focus Group Questions). Prior to focus groups, participants provided written consent and completed a brief survey on demographics and trust in medical researchers. The 90-minute discussions were audio-recorded and transcribed verbatim. Participants were compensated a $50 gift card and a meal.

#### Data analysis and establishing trustworthiness

The qualitative data coding and analysis were managed by the Vanderbilt University Qualitative Research Core. An a priori, hierarchical coding system was developed and refined using the moderator’s guide and a preliminary review of four transcripts. Major categories included: 1) opinions of presentation; 2) opinions and questions on information; 3) presenter experience; 4) Latino/African American specific issues; and 5) barriers to clinical trial and biospecimen participation. After major categories were identified, they were further divided from one to 13 subcategories, with each subcategory having additional levels of hierarchical divisions. Each category had written definitions and coding rules.

Experienced qualitative coders first established reliability in using the coding system, then independently coded the transcripts. Line-by-line coding of each transcript was done and compared, and discrepancies resolved to create a single coded transcript. Each statement was treated as a separate quote and could be assigned up to five different codes. Transcripts were combined and sorted by code. Using an iterative, inductive-deductive approach, the coded quotes were interpreted, and higher-order themes identified. This process was conducted until thematic saturation was reached. Strategies to ensure rigor included thick, rich descriptions, peer debriefing, and member checks [40]. Management of transcripts, quotations, and codes was performed using Microsoft Excel 2016 and SPSS version 24.0.

#### Iterative revision process

Starting with the modified ENACCT educational module that our team used in the previous phase, the members of the community-academic partnership team used the focus group findings to iteratively modify the module content, as well as the program delivery methods, recruitment/retention strategies, data collection, and implementation plan. A subgroup of the research team met regularly to discuss focus group findings and the PowerPoint presentation, making recommendations for changes. Then, all members of the partnership met to discuss the recommended changes and identify additional needed modifications. Following each meeting, a new iteration of the PowerPoint presentation was produced using peripheral, evidential, linguistic, and sociocultural strategies. A final meeting was held to ensure the program was culturally appropriate and incorporated all of the feedback to the best extent possible.

### Phase 3: Reading assessment with community input, 2016–2017

#### Scientific review

We identified two experts in cancer clinical trials from Vanderbilt-Ingram Cancer Center to review the content for accuracy. Selection criteria for content reviewers were: experience in cancer clinical trial research, willingness to review the PowerPoint presentation, and ability to complete the review in a timely manner. Content was deemed evidential if experts did not find inaccurate or obsolete information. Corrections were completed if identified by experts.

#### Reading level assessment

Prior to the assessment, members of the community-academic partnership reviewed the presentation and applied a linguistic strategy to replace technical terms with “lay” language and simplify the text to under a 6th grade reading level. Definitions were added to the slide or presenter notes. Reading level assessments were conducted using the Flesch Reading Ease and Flesch-Kincaid Grade Level assessments on the presentation. The Flesch Reading Ease assessment provides a score to determine level of difficulty of reading material. Material that is easier to read has higher scores, while those with lower scores are more difficult to read. The Flesch-Kincaid Grade Level assessment indicates the reading level using the U.S. grade level [41]. At this time, we were unable to identify tools to assess the reading level of the Spanish version.

#### Cognitive interviews with community members

To further evaluate the material’s comprehensiveness and ease of comprehension, we conducted 60-min interviews with two community members (one African American and one Latino) with a high school level education (i.e., a linguistic strategy). Cognitive interviews are commonly used in the development educational programs, during which community members state how they perceive and interpret materials and potential problems [42]. Specifically, we used a “think aloud” [43] approach for participants to convey their thoughts on each slide, identifying text that was difficult to understand or unclear. Slight modifications were made to problematic text.

### Phase 4: community review of new educational module, 2017

We presented the new educational presentation to two community groups to pretest the intervention’s acceptability. An African American research team member presented to the MVTCP Community Advisory Board (CAB), including primarily African American cancer survivors, community members, and representatives of cancer-focused organizations. A Latina research team member presented to a group of Latino community members within our cancer outreach network who have demonstrated interest in research studies. At the end of the presentation, participants shared their opinions aloud and completed a brief survey. Collectively, these evaluative methods gained participants’ feedback on cultural appropriateness, applying all of the cultural-targeting strategies. The research team discussed the feedback and revised the presentation accordingly.

## Results

A summary of results at each phase of program development is provided below. Using the cultural-targeting strategies recommended by Kreuter et al. (2003) [31], suggestions and revisions at each phase are organized under the following four categories: 1) peripheral; (2) evidential; (3) linguistic; and (4) sociocultural strategies. The constituent-involving strategy was incorporated with community leader and member involvement at each stage of the development process, through the community engagement process. Table 1 organizes each phase of program development by the cultural-targeting strategies (first column) and aligns the explicit suggestions for changes by participants (second column) with the specific cultural adaptations that were made to each suggestion (third column), and the relevant barriers to clinical trial participation that the changes address (fourth column).

**Table 1:** Summary of Suggestions, Changes, and Barriers to Research Culturally-Targeted at Each Phase of the Adaptation Process

**Phase II: Focus Groups**			
**Cultural Areas for Change**	**Suggestions**	**Changes**	**Targeted Barriers**
Peripheral	Increase font size on images providing cancer statistics. (AA)	Removed those images and added images with larger font and fewer statistics	-None
	Pictures need to show clinical trials are for everyone not a specific race/ethnicity (AA)	Pictures were changed to reflect all races and ethnicities	-In ability to understand information on research -Lack of information on the research process
	Change slide background color (AA)	Changed slide background color from light green to white	-None
	Increase clarity in presentation (AA, L)	Layout of slides modified to improve flow of presentation; Removed content outlining purpose of presentation; Added two takeaways to presentation	-Inability to understand information on research
Evidential	Cancer statistics should be presented by race/ethnicity and gender (AA)	Changed to overall cancer statistics for all and top cancers by race/ethnicity and gender	-Lack of information on the research process
Linguistic/Evidential	Add a video on community member experience in cancer clinical trial participation (L)	Developed videos of community members (1AA, 1 L) telling their experience in participating in a cancer clinical trial.	-Lack of information on the research process -Fear of research methods
	Need researcher perspective on research process (AA)	Developed videos of researchers (1 AA, 1 L) telling the importance of research.	-Lack of information on the research process
Linguistic	Increase clarity of who could participate in clinical trials (CHE)	Added statement that EVERYONE could participate with examples	-Lack of information on the research process
	Reduce big words to simpler language (AA)	Added definitions to presenters notes and/or replaced with simple words	-Inability to understand information on research
Sociocultural/Linguistic	Offer presentation in Spanish (L)	Translated the English version into a Spanish version	-Inability to understand information on research
Sociocultural	Provide handouts on presentation and information on ways to identify clinical trials (L)	Added handouts during presentation and added information to resource slide on where to find clinical trials	-Lack of information on the research process -Limited access to clinical trials and healthcare
	Add information culturally appealing to the community (AA, L)	-Add content on cancer (e.g., definition, etiology, risk factors), clinical trials (e.g., phases of trials and process at each phase, benefits, costs, sources to get information or register for clinical trials), and protections (e.g., Belmont Principles)	-Lack of information on the research process
**Phase III: Scientific Review**			
**Cultural Areas for Change**	**Suggestions**	**Changes**	**Targeted Barriers**
Peripheral	No Suggestions	None	-None
Evidential	Possible barriers to add include injury, travel and lodging costs, and costs to those uninsured	Added these barriers to slides along with information on patient assistance programs	-Lack of information on the research process -Concerns regarding privacy and costs
	Update presenters’ notes with information on genetic testing in biospecimens	Updated presenters’ notes with information provided on genetic testing in biospecimens	-Lack of information on the research process -Fear of research methods
Linguistic	Increase clarity on cancer treatment	Added “Cancer is more than one disease” to clarify that more than one treatment is needed to prevent cancer	-Lack of information on cancer
	Remove “safely” from “How to safely participate in clinical trials to improve cancer treatment options for your community.”	Removed the slide	-None
Sociocultural	No Suggestions	None	-None
**Phase III: Reading Level Assessment**			
**Cultural Areas for Change**	**Suggestions**	**Changes**	**Targeted Barriers**
Peripheral/Linguistic	Add visuals to compliment topic	Identified topic-appropriate visuals	-Lack of information on the research process -Fear of research methods
Evidential	Clarify difference between patient care versus research costs	Added examples of patient care and research costs	-Lack of information on the research process
	Update presenters notes to include additional information on clinical trial process	Added information on what happens at each phase of clinical trials to presenter notes	-Distrust in doctors/researchers -Lack of information on the research process -Fear of research methods
Linguistic	Use simpler term for clinical trial phases	Changed to “how a clinical trial gets to you”	-Lack of information on the research process **-**Fear of research methods
**Phase III: Cognitive Interviews**			
**Cultural Areas for Change**	**Suggestions**	**Changes**	**Targeted Barriers**
Peripheral	Aesthetics of video testimony could be improved (AA)	Decided not to refilm the video because the suggestion did not apply to the content	-Distrust in doctors/researchers -Lack of information on the research process -Fear of research methods
Evidential	Consider listing risks of being in a clinical trial (AA)	Decided not to list risk because they can vary by clinical trial; added examples of risks for a specific clinical trial to presenter notes to use if asked	-Lack of information on the research process -Fear of research methods
Linguistic	Words could be more “community friendly” (AA, L)	Minor modifications to increase clarity	-Inability to understand information on research
Sociocultural	How to participate in clinical trials if uninsured, undocumented, and speaks no English. (L)	Added to presenter notes: being undocumented as a barrier; translators in place in most instances for Spanish-speaking individuals; and patient assistance programs for those uninsured.	-Being undocumented limited the Latino community access to clinical trials
**Phase IV: Community Review**			
**Cultural Areas for Change**	**Suggestions**	**Changes**	**Targeted Barriers**
Peripheral	Change pictures to be more appealing (L)	Replaced pictures	-None
Evidential	Additional clarity needed on informed consent process (AA)	Added information to presenters’ slides on informed consent process	-Concern about privacy and costs in research participation -Distrust in doctors/researchers
Linguistic	No Comments	None	-None
Sociocultural	Use presenters from disadvantaged background (AA)	Identified CHEs representing diverse socio-economic backgrounds	-Distrust in doctors/researchers

The following abbreviations represent who made the suggestions: (AA), African American community member; (L), Latino community member; (CHE), Community Health Educator

### Community-member focus groups

Majority of focus group participants were female (78.8%), Latino (60.0%), and had an annual income of less than $25,000 (53%). Nearly one-third were married (32.9%), had a bachelor’s degree (29.4%), and were employed full-time (27.1%) (See Table 2). Five main themes emerged from these focus groups: 1) Community Perspectives on Overall Presentation; 2) Community Opinions and Questions on the Content of the Presentation; 3) Culturally Specific Issues to Participation in Cancer Clinical Trials; 4) Barriers to Clinical Trial Participation; and 5) Perspectives of Community Health Educators. Table 3 lists the definition of each theme, the sub-themes, example quotes from participants.

**Table 2:** Participant Demographics (*n* = 85)

Variable	Number	Percent
**Gender**		
Female	67	78.8
Male	18	21.2
**Race and Ethnicity**		
Black or African American	31	36.5
Hispanic/Latino	51	60.0
Other/More than one race	3	3.5
**Ethnicity**		
Hispanic/Latino	51	60.0
Not Hispanic or Latino	22	25.9
Unknown/Not Reported	3	10.6
Missing	9	3.5
**Marital Status**		
Divorced	14	16.5
Living with a partner	10	11.8
Married	28	32.9
Separated	8	9.4
Single (never married)	23	27.1
Widowed	2	2.4
**Education**		
No High School Diploma	12	14.1
GED or High School Diploma	20	23.5
Some College	15	17.6
Associates Degree	4	4.7
Bachelors Degree	25	29.4
Doctoral Degree	1	1.2
Missing	1	1.2
**Employment Status**		
Employed Full Time (32h hours per week)	23	27.1
Employed Part Time (less than 32 h per week)	18	21.2
Disability	3	3.0
Retired	4	3.5
Stay at Home	12	14.1
Volunteer	1	1.2
Other	11	12.9
Unemployed	12	14.1
**Household Income**		
Less than $10,000	34	40.0
$10,000–$14,999	5	5.9
$15,000–$24,999	6	7.1
$25,000–$34,999	13	15.3
$35,000–$49,000	6	7.1
$50,000–$74,999	4	4.7
$75,000–$99,000	1	1.2
$100,000–$149,999	4	4.7
$150,000 or more	2	2.4
Missing	10	11.8
	**Mean**	**Standard Deviation**
**Age**	42	12.5

**Table 3:** Emerging illustrating themes on participants views towards cancer clinical trials and the education program

Theme	Subthemes	Examples of Participant Statements
Community Perspectives on the Overall Presentation **Definition**: Describes the participants’ thoughts on the presentation itself (e.g., aesthetics and data presentation)	-Statistics -Order -Images/Figures/Videos -Materials -Length -Clarity -Audience size -Testimonial -Location -Other	“... the information is very clear. It does not contain any scientific or elevated information that is difficult to understand. I think that most of the population can understand it.” **(Clarity, Latino)** “Bigger, 50 to 100 people, they begin to look at the ceiling. In that way, you have the control of the group. Bigger groups, you lose control of the group.” **(Size of Audience, Latino)** “... the language was okay. I thought it was simple enough. I can’t imagine how more simpler you could explain that. I would be infuriated if you tried to break it down anymore, but that’s a personal thing...” **(Clarity, African American)** “… the color, I don’t think that works for a lot of people ... you can dismiss it. **(Other, African American)**
Community Opinions and Questions on the Content of the Presentation **Definition:** Describes the participants’ thoughts and questions on the information provided in the presentation. This applies to data, topics, etc.	-Too Specific -Not enough information/specificity -Relevance to population -Benefits of clinical trials/biospecimens -Cause/ background/ prevention -Financial -Personal -Clinical Trials/ Biospecimen Process -Purpose of presentation -Participation -Increased Awareness -Respectful/appropriate -Children	”Do we have bad habits that we are not aware of and that is contributing to so much cancer, for example, no??” **(Not Enough Information, Latino)** “...a definition of what a clinical trial is.” **(Not Enough Information, Latino)** “Put positive stuff behind it just to show numbers and stuff. You know, like you got like 45% of people having success in going through clinical trials or something like that.” **(Not Enough Information, African American)** “Maybe if you could ... if somebody would come forward and do a testimony ... if they’ve been in that type of situation or something going on with them with cancer or something, maybe they would come and tell they story, if somebody would come forward.” **(Not Enough Information, African American)**
Culturally Specific Issues to Participation in Cancer Clinical Trials **Definition:** Distinguishes specific cultural concerns for Latinos and African Americans	-Latino specific issues -African American specific issues	**“**Clearly, there’s an understanding that there’s a huge mistrust, particularly in the African American community, and so when you see information about HIPAA regulations or that kind of thing, that is a glimmer of hope for many people.” **(Mistrust in Research, African American)** “Your lifestyle and your diet, that’s why ... (inaudible) ... and the very unhealthy way they eat. They are brought up with that. What they idolize is not healthy. I mean, how often do you hear ... (inaudible) ... eating healthy, wheat bread? I eat Kool-Aid, hot chips, corn flakes …” **(Diet, African American)** **“**I also think it’s because of fear and because many people don’t know how to read. They don’t have this ability that we have.” **(Fear of Research Methods, Latino)** **“**I think it’s also trying to get us (the Spanish community) to participate more, because there is little information out there for us to go …” **(Language Barrier, Latino)**
Barriers to Clinical Trial Participation **Definition:** Participant mentions barriers to clinical trial and biospecimen participation	-Language -Lack of Information -Privacy -Fear of research methods -Cost -Immigration Status -Mistrust in doctors	“Talk more about the fear we have to participate, because I hesitate because I think, well, what are the risks or side effects?” That is the biggest fear. People don’t want to feel like a guinea pig.” **(Fear of Research Methods, Latino)** “I think lack of information is the major problem. There are a lot of people that are afraid of what is going to happen to them, side effects, because a lot of people don’t like to take medicine, including me. Also, about insurance and all that, that also should be clear ....” **(Lack of Information, Latino)** “I got a problem with these clinical trials. They say they tell you about the risks and all this and all that, but they ain’t ... like she said, she’s got asthma, but then they gave her an inhaler that had something, you know, that she was allergic to and she probably didn’t even know about it.” **(Mistrust in Doctors, African American)** ”... A lot of times it’s just lack of knowledge. People go through everyday thinking and they’re not plugged in, or they don’t have the information to know these things are out there ... (inaudible), and a lot of people don’t know it’s there.” **(Lack of Information, African American)**
Perspectives of Community Health Educators **Definition:** Addresses the opinions of the CHEs that delivered the original module in the previous project phase.	-Comfortable/satisfied -Discomfort -Group dynamic -Importance of community -Recruitment	”I felt very comfortable with presenting the slides. I thought the slides were concise.” **(Presenter)** “And they came and did some tweaking, and then we got back together. So, we had input all along the way.” **(Presenter)** ... I’m not sure that people know that there is an opportunity for them to be a part of this peer education process [Be a CHE]. Now, if we could find ways to get that out there, just like getting the word out, that would increase the number of people involved in the communities that you are trying to pull into this.” **(Presenter)**

#### Community perspectives on the overall presentation

Overall, participants perceived the presentation was clear, images appropriate, and language simple. However, participants suggested changing the order of some slides for smoother transitions throughout the presentation. Additional suggestions were to enhance blurred images, present cancer statistical information by race/ethnicity and gender, add a testimonial on experience with clinical trial participation, and end with a takeaway message. The target audience of African Americans and Latinos aged 18 and up was deemed appropriate, yet they also encouraged all ages to view the presentation. The small audience size (12–15 people) for the presentation was acceptable, creating discussion and keeping focus. For location, they said it was important to have an enclosed space to limit distractions for participants and the presenter. Lastly, participants stated they needed presentation handouts and an information sheet on how to enroll in clinical trials.

#### Community opinions and questions on the content of the presentation

Participants believed the presentation increased awareness and was appropriate and respectful. Yet, they perceived the information too specific at times and wanted more general information. They more general information (e.g., statistics), and pictures that were diverse and not reflecting one racial/ethnic group. Participants also believed the clarity of the presentation could be improved by adding information on cancer biology and risk factors, and more details on the clinical trial and biospecimen collection process. Furthermore, it was suggested to target content to Latino and African American audiences separately while adding personal testimonials on participant clinical trial experiences. Specifically, images and testimonials should match race or ethnicity of target audience.

#### Culturally specific issues to participation in Cancer clinical trials

Both groups emphasized the history of abuse in medical research and being “lied to” by medical researchers and/or physicians. Many participants conveyed high levels of distrust in providers/researchers. The cancer disparity among African Americans was discussed and culture (e.g., disbelief in the cancer disparity) contributed to low cancer clinical trial participation rates among participants. For Latinos, being Spanish-speaking was a specific issue, creating fear by not understanding the material presented for cancer clinical trial participation.

#### Barriers to clinical trial participation

Top barriers were lack of information on the research process, followed by fear of research methods, and distrust in doctors/researchers. Additional barriers cited by participants included concerns regarding privacy and costs in research participation. A specific barrier to the Latino community was immigration status. Being “undocumented” limited their access to clinical trials and healthcare. A specific barrier to the African American community was limited access to clinical trials and healthcare.

#### Perspectives of community health educators

Overall, educators felt confident and comfortable in presenting the material after training. They understood their role and how important it was for them to teach the community about cancer, clinical trials, and biospecimen research. They appreciated the opportunity to assist in adapting the presentation and providing input on content and aesthetics based on their prior experiences. Lastly, their recommendation was to recruit community leaders with rapport in communities to serve as future educators, making the program more targeted.

### Cultural adaptations using focus group findings

Based on the focus group findings, suggestions for changes and barriers to clinical trial participation, we applied the four cultural-targeting strategies to perform the adaptations listed in Table 1. From the peripheral perspective, the slide background color was changed from light green to white to increase the aesthetic appeal of the presentation. Pictures were changed to reflect all races and ethnicities, indicating clinical trials were for everyone not a specific race or ethnicity. The layout of the slides were altered to be more visually appealing, and the slide order was modified to improve the flow of the presentation. The targeted, evidential statements regarding cancer epidemiology (i.e., incidence, mortality, and survival rates) among both racial/ethnic groups were altered to reflect general cancer incidence and mortality rates and a list of top 3 cancers by race/ethnicity and gender. Furthermore, video testimonials were added to the presentation. In applying the linguistic strategy, we hired a translator to translate the English version of the presentation to a second Spanish version. For video testimonials, the Latino community member and the researcher each made two videos on the importance of research (2 English and 2 Spanish versions) plus two videos with and an African American community member and researcher (English version only). Images and graphics that were not understood by community members were removed. Last, from the sociocultural perspective, we ensured the program fit the specific needs of the participants of each cultural group by adding content on facts related to cancer (e.g., definition, etiology, and risk factors), clinical trials (e.g., phases of clinical trials and process at each phase, benefits, costs, sources to get information or register for clinical trials), and protections (e.g., Belmont Principles). We removed the content outlining the purpose of the presentation and added two takeaways from the presentation.

### Program finalization

#### Scientific review

Clinical trial experts perceived the presentation as “great”, particularly the content, format, and diversity in images. Minor modifications were made to increase clarity from the linguistic and evidential perspectives. For example, we added additional costs (i.e., travel and lodging, injury, out-of-pocket expenses) to clinical trial participation among these groups). We added or removed text to increase clarity of statements. Additional information (e.g., the collection of specimens for genetic testing for targeted treatments) was provided in the speaker notes to ensure presenters have sufficient information to answer potential questions posed by community members. No changes were suggested relative to the peripheral and sociocultural categories.

#### Reading level assessment

The Flesch Reading Ease test value was 68.2 and the Flesch-Kincaid Grade Level was 5.5, which met our target level. In applying linguistic and peripheral strategies, the language was simplified further. Also, plain language principles were used by adding images to increase clarity of the content [44].

#### Cognitive interview

Results of the community member interviews indicated only minor modifications in wording were needed for clarity under the linguistic strategy. For the evidential strategy, the participants suggested adding a list of risks in being in a clinical trial, which we added only in the presenter notes for reference when answering questions, since they vary across trials. Socioculturally, we added information on how to participate in a clinical trial if undocumented, uninsured, and speaks no English.

### Community review of new educational module

Overall, participants had positive reviews on the presentation. They perceived it was informative, concise, organized and timely. The videos that provide the community member and researcher perspectives and/or experiences on cancer clinical trials were well-received. No participants found the material to be culturally insensitive. Members perceived presenters were knowledgeable on the topic, engaging, prepared, well-spoken, and effective in responding to the audience questions and comments. However, some participants perceived pertinent information was missing on the topic (e.g., additional information on cancer prevention and clinical trial phases) or the presentation was lengthy. See Table 4 for examples of participant feedback. Using their feedback, pictures were updated (i.e., peripheral strategy), additional information was added to the presenters notes on the informed consent process (evidential strategy), and CHEs were identified from all socio-economical statuses to provide the presentation (sociocultural strategy).

**Table 4:** Feedback and Suggestions from Community Review

	Positive Feedback	Negative Feedback
Meharry Vanderbilt Tennessee Cancer Partnership Community Advisory Board (*n* = 12)	**Presentation** • Length is good • Content brief • Content informative • Balance between texts, figures, and images	**Presentation** • Lengthy • Informed consent process unclear on ethics slide • Community member video not appealing
Latino Community Partner Group (*n* = 11)	**Presentation** • Content comprehendible • Videos are good **Presenter** • Presenter prepared • Presenter understandable	**Presentation** • Too many slides • Too much detail • “Missing information on cancer prevention”

### Final program

Through this formative research process, we developed a new, 25-slide educational program entitled, “Clinical Trials: What’s In It For Us?”. The final program consisted of two presentations designed to be culturally-appropriate for African Americans and Latinos, plus video testimonials on cancer clinical trials from community members and researchers who were African American and Latino (in English). There was an additional Spanish version available for the presentation and testimonial videos for the Latino community. While nearly all content was changed from the ENACCT module, we added a note acknowledging ENACCT as the source on relevant slides with any specific content from the original ENACCT slides.

## Discussion

Our goal was to conduct a formative research process to develop a culturally-appropriate, cancer clinical trial educational program that improves knowledge, attitudes, willingness, and ultimately participation in cancer clinical trials and biospecimen collection. We intricately interweaved community engagement throughout the multi-layered, development process to target this program, offering a different perspective for the program. Community involvement through formative research and message targeting have demonstrated effectiveness in promoting behavior change [45, 46]. This formative research process serves as an example on how to apply CEnR principles and cultural-targeting strategies to develop culturally-appropriate program to increase cancer clinical trials participation among African Americans and Latinos.

Past research has applied CEnR principles to develop clinical trials educational programs, but they were limited to short-term involvement of community stakeholders in a consulting role [21–24]. Reflecting Wilkins’ et al. (2018) framework for stakeholder engagement in health research [47], we used community members of all levels at varying extents of involvement - team members (CBO leaders), advisory groups (MVTCP advisory board), and reviewers/consultants (CHEs, community members) - to adapt our program for the African American and Latino communities. This was an innovative, collaborative method which listened to the community’s *voice* and promoted consensus building throughout the process. We specifically sought input to make the program relevant, accurate, and culturally appropriate with effective delivery methods. Furthermore, our CBO leaders served as co-authors to disseminate our work to academicians.

Previous studies have used formative research to develop culturally-targeted interventions focused on other topics [48, 49], and the use of community engagement in formative research has increased over the years [37, 50]. Both approaches have been found effective; however, as we have noted, few examples exist in the application of CEnR principles to culturally target programs in the formative research process [51]. For example, Vastine et al. (2005) presented a model to develop a culturally appropriate dietary intervention in a formative research process using stakeholder participation [51, 52]. Yet, the existing literature provides limited guidance on how to apply CEnR principles and cultural-targeting strategies in formative research to ensure a program is culturally appropriate, with no previous examples identified on the topic of clinical trials education. We have demonstrated the incorporation of CEnR during a formative research process to design a culturally target intervention aimed to increase participation in cancer clinical trials among Latinos and African Americans. This formative research and engagement process can be used to guide the development other programs seeking to produce behavior change to improve health outcomes.

### Strengths and limitations

A major strength of this process was utilizing our existing CEnR infrastructure to apply a multi-layered, CEnR approach and cultural-targeting strategies to develop this program. We empowered the African American and Latino communities to advocate for their educational needs on cancer and the clinical trial process, increasing their ability to make informed decisions on cancer clinical trial participation. They can also serve as an educational resource to other community members, increasing awareness and acceptability of cancer clinical trials, and possibly clinical trials in general. There are a few limitations. Due to using an iterative, multi-layered approach for program development, our timeline was extensive to incorporate community feedback. For example, focus group feedback led to the development of two presentations, one for the African American community and one for the Latino community. However, this timeline could be shortened if feedback already exists and if only one presentation is needed. Second, there are logistical barriers that can inhibit clinical trial participation (e.g., lack of insurance) that the educational program cannot address, and thus, could limit the effectiveness of this program on clinical trial participation outcomes when implemented.

### Next steps

The next step of our partnership was to implement our cancer clinical trials educational program in a pilot study. Our team is evaluating the programs’ impact on participant knowledge, attitudes, trust, and willingness related to cancer clinical trials participation, which will be reported in a future manuscript.

## Conclusion

We developed a culturally-appropriate, cancer clinical trial education program using a multi-layered, iterative formative research process while applying CEnR principles and cultural-targeting strategies. It allowed for identifying logistical barriers and fit the needs of the target audience, increasing chances of program acceptability and effectiveness. This program can be adopted by existing CBOs and institutions to educate these communities on cancer and clinical trials. This offers the potential to improve knowledge, attitudes, and behavior related to cancer clinical trials in the long term.

## Supplementary information

**Additional file 1.** Focus Group Guide.

## Data Availability

The data are not publicly available due to them containing information that could compromise research participant privacy/consent.

## Abbreviations

CBO: Community Based Organization
CEnR: Community Engaged Research
CHE: Community Health Educator
ENACCT: Education Network to Advance Cancer Clinical Trials
MMC: Meharry Medical College
MVA: Meharry-Vanderbilt Alliance
MWCHC: Matthew Walker Comprehensive Health Center
PCC: Progresso Community Center
TSU: Tennessee State University
VICC: Vanderbilt Ingram Cancer Center
