# Supplementary material for: Formative research to design a culturally-appropriate cancer clinical trial education program to increase participation of African American and Latino communities
Source: BMC Public Health. 2020 Jun 3;20:840. doi: 10.1186/s12889-020-08939-4 (PMC7268329; doi:10.1186/s12889-020-08939-4)
Supplement: Supplementary file 1 — Additional file 1. Focus Group Guide. [file 12889_2020_8939_MOESM1_ESM.docx]

**Supplementary File A**

**Community Member Focus Group Questions**

1) To start out the discussion, what is your overall impression of the workshop?

2) What would you say were the key take-home messages from the workshop?

3) How clear was the information in the workshop?

• Were there any parts of the workshop that were not clear or were confusing?

Please give us specific examples.

4) Who do you think are the target audiences for the workshop?

5) Thinking about the information, language, and images used in the workshop, do you feel

it was appropriate for your age, gender, racial/ethnic group, and cultural background?

Please explain.

• How respectful and sensitive to your feelings and beliefs was it? Please explain.

• Was there anything in the presentation that you felt was offensive or insensitive

or made you feel uncomfortable? Please give us specific examples.

6) What could be improved to make sure the workshop is appropriate for people from

various different backgrounds and walks of life?

7) What other suggestions do you have to improve the information in the workshop and the

way it is presented?

8) Is there anything else we haven’t talked about yet that you would like to share or

suggest?

**Previously Trained Health Educator Focus Group Questions**

Content areas: level of comfort in delivering the message, level of understanding of the material/content, fluidity of the presentation, receptivity of the message by the audience

1) What was your reaction to the training?

• How well do you feel you understood the content in the presentation?

• What would have made the training better?

2) Describe your experience delivering the training.

• How comfortable were you with the time given to present the material?

• How comfortable did you feel delivering the message to the audience?

• Were there areas during the presentation when the audience seemed confused

or unsure about the message?

• What changes would you suggest to make it clearer?

• Were there areas during the presentation when the audience seemed bored or

disinterested?

• What changes would you suggest to make the presentation more appealing or

interesting?

• What types of questions did your audience ask? Were any of their questions

culture or community specific?
